# *N*-(2-Hydroxy­ethyl)-1,8-naphthalimide

**DOI:** 10.1107/S1600536809015621

**Published:** 2009-05-07

**Authors:** Jie Sun, Ai-lin Yuan, Hai-Bo Wang, Jie Sun

**Affiliations:** a College of Food Science and Light Industry, Nanjing University of Technology, Xinmofan Road No. 5 Nanjing, Nanjing 210009, People’s Republic of China

## Abstract

In the mol­ecule of the title compound, C_14_H_11_NO_3_, the naphthalimide ring system is nearly planar (r.m.s. deviation 0.0139 Å). In the crystal structure, inter­molecular O—H⋯O hydrogen bonds link the mol­ecules into centrosymmetric dimers forming *R*_2_^2^(14) ring motifs. π–π contacts between the naphthalimide rings [centroid–centroid distances = 3.648 (3), 3.783 (3), 3.635 (3), 3.722 (3) and 3.755 (3) Å] may further stabilize the structure.

## Related literature

For a related structure, see: Prezhdo *et al.* (2007 ▶). For bond-length data, see: Allen *et al.* (1987 ▶). For ring-motifs, see: Bernstein *et al.* (1995 ▶).
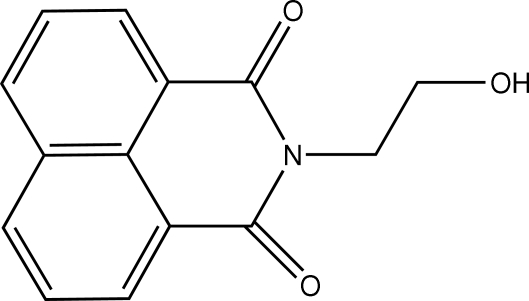


## Experimental

### 

#### Crystal data

C_14_H_11_NO_3_
*M* *_r_* = 241.24
Triclinic, 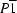

*a* = 7.5480 (15) Å
*b* = 8.8300 (18) Å
*c* = 10.101 (2) Å
α = 96.760 (19)°
β = 109.94 (3)°
γ = 114.60 (3)°
*V* = 548.2 (3) Å^3^
*Z* = 2
Mo *K*α radiation
μ = 0.10 mm^−1^
*T* = 294 K
0.30 × 0.20 × 0.10 mm

#### Data collection

Enraf–Nonius CAD-4 diffractometer
Absorption correction: ψ scan (North *et al.*, 1968 ▶) *T*_min_ = 0.970, *T*_max_ = 0.990
2159 measured reflections
1995 independent reflections
1330 reflections with *I* > 2σ(*I*)
*R*_int_ = 0.023
3 standard reflections frequency: 120 min intensity decay: 1%

#### Refinement

*R*[*F* ^2^ > 2σ(*F* ^2^)] = 0.054
*wR*(*F* ^2^) = 0.208
*S* = 1.00
1995 reflections
164 parameters
H-atom parameters constrained
Δρ_max_ = 0.29 e Å^−3^
Δρ_min_ = −0.31 e Å^−3^

### 

Data collection: *CAD-4 Software* (Enraf–Nonius, 1989 ▶); cell refinement: *CAD-4 Software*; data reduction: *XCAD4* (Harms & Wocadlo, 1995 ▶); program(s) used to solve structure: *SHELXS97* (Sheldrick, 2008 ▶); program(s) used to refine structure: *SHELXL97* (Sheldrick, 2008 ▶); molecular graphics: *ORTEP-3 for Windows* (Farrugia, 1997 ▶) and *PLATON* (Spek, 2009 ▶); software used to prepare material for publication: *SHELXL97* and *PLATON*.

## Supplementary Material

Crystal structure: contains datablocks global, I. DOI: 10.1107/S1600536809015621/hk2674sup1.cif

Structure factors: contains datablocks I. DOI: 10.1107/S1600536809015621/hk2674Isup2.hkl

Additional supplementary materials:  crystallographic information; 3D view; checkCIF report

## Figures and Tables

**Table 1:** Hydrogen-bond geometry (Å, °)

*D*—H⋯*A*	*D*—H	H⋯*A*	*D*⋯*A*	*D*—H⋯*A*
O1—H1*A*⋯O2^i^	0.82	1.97	2.771 (4)	165

Symmetry code: (i) 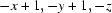.

**Fig. 1.**
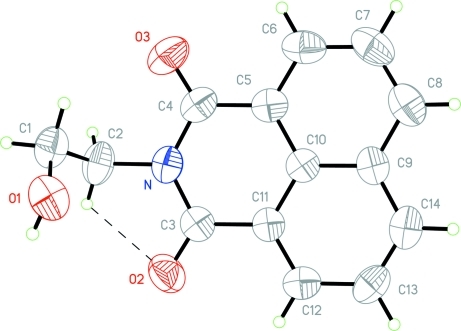
The molecular structure of the title molecule, with the atom-numbering scheme. Displacement ellipsoids are drawn at the 50% probability level. Hydrogen bond is shown as dashed line.

**Fig. 2.**
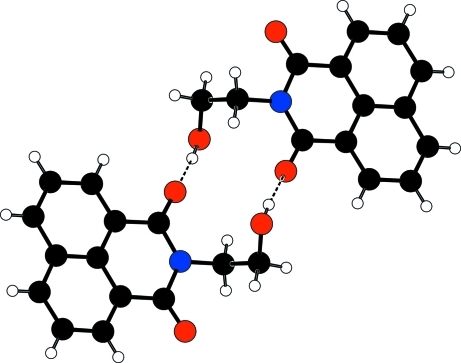
A partial packing diagram of the title compound. Hydrogen bonds are shown as dashed lines.

## supplementary crystallographic information

### Comment

As part of our ongoing studies on N-substituted 1,8-naphthalimides (Prezhdo *et al.*,  2007), we report herein the crystal structure of the title compound.

In the molecule of the title compound (Fig 1), the bond lengths (Allen *et al.*,  1987) and angles are within normal ranges. Rings A (N/C3-C5/C10/C11), B (C5-C10) and C (C9-C14) are, of course, planar, and they are oriented at dihedral angles of A/B = 1.79 (3), A/C = 1.14 (3) and B/C = 1.00 (3) °. So, they are nearly coplanar. Intramolecular C-H···O interaction (Table 1) results in the formation of a five-membered ring D (O2/N/C2/C3/H2A), having envelope conformation, with atom H2A displaced by -0.302 (3) Å from the plane of the other ring atoms.

In the crystal structure, intermolecular O-H···O hydrogen bonds (Table 1) link the molecules into centrosymmetric dimers forming R_2_^2^(14) ring motifs (Fig. 2) (Bernstein *et al.*, 1996), in which they may be effective in the stabilization of the structure. The π–π contacts between the naphthalimide rings, Cg1—Cg1^i^, Cg1—Cg2^i^, Cg1—Cg3^ii^, Cg2—Cg3^ii^ and Cg3—Cg3^ii^ [symmetry codes: (i) 1 - x, 1 - y, 1 - z, (ii) 2 - x, 1 - y, 1 - z, where Cg1, Cg2 and Cg3 are centroids of the rings A (N/C3-C5/C10/C11), B (C5-C10) and C (C9-C14), respectively] may further stabilize the structure, with centroid-centroid distances of 3.648 (3), 3.783 (3), 3.635 (3), 3.722 (3) and 3.755 (3) Å, respectively.

### Experimental

For the preparation of the title compound, 1,8-naphthalic anhydride (1.98 g, 0.01 mol) and 2-aminoethanol (0.02 mol) were mixed with acetic acid (50 ml). The reaction mixture was refluxed for 8 h, and then poured into cold water. The resulting solids were filtered off. The solid products were boiled with an aqueous solution of sodium bicarbonate (10%, 50 ml) for 20 min, and the insoluble solid residues were dried in vacuo. Column chromatography on aluminium oxide with the C_6_H_6_ eluent gave light-brown solution. Crystals suitable for X-ray analysis were obtained by slow evaporation of an acetone solution (yield; 96%, m.p. 413 K).

### Refinement

H atoms were positioned geometrically, with O-H = 0.82 Å (for OH) and C-H = 0.93 and 0.97 Å for aromatic and methylene H, respectively, and constrained to ride on their parent atoms, with U_iso_(H) = xU_eq_(C,O), where x = 1.5 for OH H and x = 1.2 for all other H atoms.

### Crystal data

**Table:**

C_14_H_11_NO_3_	*Z* = 2
*M**_r_* = 241.24	*F*(000) = 252
Triclinic, *P*1	*D*_x_ = 1.461 Mg m^−^^3^
Hall symbol: -P 1	Melting point: 413 K
*a* = 7.5480 (15) Å	Mo *K*α radiation, λ = 0.71073 Å
*b* = 8.8300 (18) Å	Cell parameters from 25 reflections
*c* = 10.101 (2) Å	θ = 10–13°
α = 96.760 (19)°	µ = 0.10 mm^−^^1^
β = 109.94 (3)°	*T* = 294 K
γ = 114.60 (3)°	Block, green
*V* = 548.2 (3) Å^3^	0.30 × 0.20 × 0.10 mm

### Data collection

**Table:**

Enraf–Nonius CAD-4 diffractometer	1330 reflections with *I* > 2σ(*I*)
Radiation source: fine-focus sealed tube	*R*_int_ = 0.023
graphite	θ_max_ = 25.3°, θ_min_ = 2.3°
ω/2θ scans	*h* = 0→9
Absorption correction: ψ scan (North *et al.*, 1968)	*k* = −10→9
*T*_min_ = 0.970, *T*_max_ = 0.990	*l* = −12→11
2159 measured reflections	3 standard reflections every 120 min
1995 independent reflections	intensity decay: 1%

### Refinement

**Table:**

Refinement on *F*^2^	Secondary atom site location: difference Fourier map
Least-squares matrix: full	Hydrogen site location: inferred from neighbouring sites
*R*[*F*^2^ > 2σ(*F*^2^)] = 0.054	H-atom parameters constrained
*wR*(*F*^2^) = 0.208	*w* = 1/[σ^2^(*F*_o_^2^) + (0.1*P*)^2^ + 0.4*P*] where *P* = (*F*_o_^2^ + 2*F*_c_^2^)/3
*S* = 1.00	(Δ/σ)_max_ < 0.001
1995 reflections	Δρ_max_ = 0.29 e Å^−^^3^
164 parameters	Δρ_min_ = −0.31 e Å^−^^3^
0 restraints	Extinction correction: *SHELXL97* (Sheldrick, 2008), Fc^*^=kFc[1+0.001xFc^2^λ^3^/sin(2θ)]^-1/4^
Primary atom site location: structure-invariant direct methods	Extinction coefficient: 0.035 (10)

### Special details

**Table:**

Geometry. All esds (except the esd in the dihedral angle between two l.s. planes) are estimated using the full covariance matrix. The cell esds are taken into account individually in the estimation of esds in distances, angles and torsion angles; correlations between esds in cell parameters are only used when they are defined by crystal symmetry. An approximate (isotropic) treatment of cell esds is used for estimating esds involving l.s. planes.
Refinement. Refinement of F^2^ against ALL reflections. The weighted R-factor wR and goodness of fit S are based on F^2^, conventional R-factors R are based on F, with F set to zero for negative F^2^. The threshold expression of F^2^ > 2sigma(F^2^) is used only for calculating R-factors(gt) etc. and is not relevant to the choice of reflections for refinement. R-factors based on F^2^ are statistically about twice as large as those based on F, and R- factors based on ALL data will be even larger.

### Fractional atomic coordinates and isotropic or equivalent isotropic displacement parameters (Å^2^)

**Table:**

	*x*	*y*	*z*	*U*_iso_*/*U*_eq_	
O1	0.4860 (4)	0.2063 (3)	−0.0242 (2)	0.0624 (7)	
H1A	0.4763	0.2711	−0.0749	0.094*	
O2	0.6221 (4)	0.5936 (3)	0.1937 (3)	0.0677 (8)	
O3	0.2875 (4)	0.1029 (3)	0.3070 (3)	0.0729 (8)	
N	0.4553 (4)	0.3481 (3)	0.2496 (3)	0.0480 (7)	
C1	0.3020 (6)	0.1341 (5)	0.0027 (4)	0.0599 (9)	
H1B	0.3052	0.0455	0.0504	0.072*	
H1C	0.1746	0.0767	−0.0913	0.072*	
C2	0.2818 (5)	0.2660 (5)	0.0982 (4)	0.0610 (10)	
H2A	0.2828	0.3566	0.0522	0.073*	
H2B	0.1439	0.2081	0.1026	0.073*	
C3	0.6193 (5)	0.5166 (4)	0.2858 (3)	0.0472 (8)	
C4	0.4392 (5)	0.2495 (4)	0.3487 (4)	0.0509 (8)	
C5	0.6124 (5)	0.3314 (4)	0.5006 (3)	0.0464 (8)	
C6	0.6103 (6)	0.2394 (5)	0.6016 (4)	0.0601 (10)	
H6A	0.4975	0.1263	0.5743	0.072*	
C7	0.7759 (7)	0.3145 (5)	0.7442 (4)	0.0677 (11)	
H7A	0.7726	0.2510	0.8114	0.081*	
C8	0.9433 (6)	0.4804 (5)	0.7870 (4)	0.0601 (9)	
H8A	1.0531	0.5286	0.8826	0.072*	
C9	0.9504 (5)	0.5784 (4)	0.6872 (3)	0.0450 (7)	
C10	0.7830 (5)	0.5027 (4)	0.5413 (3)	0.0407 (7)	
C11	0.7899 (5)	0.5973 (4)	0.4390 (3)	0.0423 (7)	
C12	0.9566 (5)	0.7653 (4)	0.4817 (4)	0.0494 (8)	
H12A	0.9611	0.8282	0.4142	0.059*	
C13	1.1206 (5)	0.8421 (4)	0.6277 (4)	0.0560 (9)	
H13A	1.2317	0.9562	0.6562	0.067*	
C14	1.1181 (5)	0.7516 (5)	0.7266 (4)	0.0536 (9)	
H14A	1.2283	0.8041	0.8222	0.064*	

### Atomic displacement parameters (Å^2^)

**Table:**

	*U*^11^	*U*^22^	*U*^33^	*U*^12^	*U*^13^	*U*^23^
O1	0.0681 (16)	0.0792 (17)	0.0581 (15)	0.0486 (14)	0.0293 (12)	0.0251 (12)
O2	0.0783 (17)	0.0742 (17)	0.0564 (15)	0.0431 (14)	0.0243 (13)	0.0308 (13)
O3	0.0595 (16)	0.0526 (15)	0.0854 (19)	0.0114 (13)	0.0330 (14)	0.0078 (13)
N	0.0442 (15)	0.0521 (15)	0.0468 (15)	0.0266 (13)	0.0177 (12)	0.0061 (12)
C1	0.057 (2)	0.059 (2)	0.053 (2)	0.0252 (17)	0.0200 (16)	0.0039 (16)
C2	0.0468 (19)	0.068 (2)	0.055 (2)	0.0298 (17)	0.0112 (16)	0.0000 (17)
C3	0.0512 (19)	0.0525 (18)	0.0517 (19)	0.0341 (16)	0.0260 (15)	0.0161 (15)
C4	0.0480 (19)	0.0451 (18)	0.063 (2)	0.0226 (16)	0.0310 (16)	0.0069 (15)
C5	0.0514 (18)	0.0469 (17)	0.0566 (19)	0.0302 (15)	0.0327 (16)	0.0148 (15)
C6	0.078 (2)	0.056 (2)	0.075 (3)	0.0383 (19)	0.054 (2)	0.0285 (18)
C7	0.095 (3)	0.081 (3)	0.063 (2)	0.057 (2)	0.049 (2)	0.039 (2)
C8	0.073 (2)	0.081 (3)	0.051 (2)	0.052 (2)	0.0338 (18)	0.0238 (18)
C9	0.0469 (17)	0.0588 (19)	0.0434 (17)	0.0359 (16)	0.0232 (14)	0.0123 (14)
C10	0.0421 (16)	0.0460 (16)	0.0483 (17)	0.0291 (14)	0.0260 (14)	0.0120 (13)
C11	0.0455 (17)	0.0447 (16)	0.0463 (17)	0.0296 (14)	0.0214 (14)	0.0119 (13)
C12	0.0551 (19)	0.0443 (17)	0.062 (2)	0.0293 (15)	0.0328 (16)	0.0171 (15)
C13	0.0434 (18)	0.0478 (18)	0.068 (2)	0.0186 (15)	0.0238 (17)	0.0021 (17)
C14	0.0481 (18)	0.065 (2)	0.0490 (19)	0.0339 (17)	0.0190 (15)	0.0025 (16)

### Geometric parameters (Å, °)

**Table:**

O1—C1	1.403 (4)	C6—C7	1.391 (5)
O1—H1A	0.8200	C6—H6A	0.9300
O2—C3	1.216 (4)	C7—C8	1.366 (5)
O3—C4	1.214 (4)	C7—H7A	0.9300
N—C2	1.470 (4)	C8—C9	1.404 (5)
N—C3	1.383 (4)	C8—H8A	0.9300
N—C4	1.404 (4)	C9—C10	1.418 (4)
C1—C2	1.515 (5)	C9—C14	1.414 (5)
C1—H1B	0.9700	C10—C11	1.405 (4)
C1—H1C	0.9700	C11—C12	1.376 (4)
C2—H2A	0.9700	C12—C13	1.410 (5)
C2—H2B	0.9700	C12—H12A	0.9300
C3—C11	1.476 (4)	C13—C14	1.351 (5)
C4—C5	1.474 (5)	C13—H13A	0.9300
C5—C6	1.378 (5)	C14—H14A	0.9300
C5—C10	1.409 (4)		
			
C1—O1—H1A	109.5	C5—C6—H6A	119.8
C3—N—C4	124.6 (3)	C7—C6—H6A	119.8
C3—N—C2	118.3 (3)	C8—C7—C6	120.9 (3)
C4—N—C2	117.1 (3)	C8—C7—H7A	119.6
O1—C1—C2	114.2 (3)	C6—C7—H7A	119.6
O1—C1—H1B	108.7	C7—C8—C9	120.4 (3)
C2—C1—H1B	108.7	C7—C8—H8A	119.8
O1—C1—H1C	108.7	C9—C8—H8A	119.8
C2—C1—H1C	108.7	C8—C9—C14	122.6 (3)
H1B—C1—H1C	107.6	C8—C9—C10	119.2 (3)
N—C2—C1	113.5 (3)	C14—C9—C10	118.2 (3)
N—C2—H2A	108.9	C11—C10—C5	120.9 (3)
C1—C2—H2A	108.9	C11—C10—C9	120.0 (3)
N—C2—H2B	108.9	C5—C10—C9	119.1 (3)
C1—C2—H2B	108.9	C12—C11—C10	119.9 (3)
H2A—C2—H2B	107.7	C12—C11—C3	120.0 (3)
O2—C3—N	120.7 (3)	C10—C11—C3	120.1 (3)
O2—C3—C11	121.9 (3)	C11—C12—C13	120.1 (3)
N—C3—C11	117.4 (3)	C11—C12—H12A	119.9
O3—C4—N	119.8 (3)	C13—C12—H12A	119.9
O3—C4—C5	123.0 (3)	C14—C13—C12	120.7 (3)
N—C4—C5	117.2 (3)	C14—C13—H13A	119.7
C6—C5—C10	120.1 (3)	C12—C13—H13A	119.7
C6—C5—C4	120.1 (3)	C13—C14—C9	121.1 (3)
C10—C5—C4	119.8 (3)	C13—C14—H14A	119.4
C5—C6—C7	120.4 (3)	C9—C14—H14A	119.4
			
C3—N—C2—C1	103.2 (4)	C4—C5—C10—C11	0.4 (4)
C4—N—C2—C1	−78.1 (4)	C6—C5—C10—C9	−0.4 (4)
O1—C1—C2—N	−64.6 (4)	C4—C5—C10—C9	−178.9 (2)
C4—N—C3—O2	180.0 (3)	C8—C9—C10—C11	−178.6 (3)
C2—N—C3—O2	−1.4 (4)	C14—C9—C10—C11	1.9 (4)
C4—N—C3—C11	0.5 (4)	C8—C9—C10—C5	0.7 (4)
C2—N—C3—C11	179.1 (2)	C14—C9—C10—C5	−178.9 (2)
C3—N—C4—O3	179.1 (3)	C5—C10—C11—C12	179.1 (2)
C2—N—C4—O3	0.5 (4)	C9—C10—C11—C12	−1.6 (4)
C3—N—C4—C5	−1.2 (4)	C5—C10—C11—C3	−1.1 (4)
C2—N—C4—C5	−179.9 (2)	C9—C10—C11—C3	178.1 (2)
O3—C4—C5—C6	2.0 (5)	O2—C3—C11—C12	1.0 (4)
N—C4—C5—C6	−177.7 (3)	N—C3—C11—C12	−179.6 (3)
O3—C4—C5—C10	−179.6 (3)	O2—C3—C11—C10	−178.8 (3)
N—C4—C5—C10	0.8 (4)	N—C3—C11—C10	0.7 (4)
C10—C5—C6—C7	0.1 (5)	C10—C11—C12—C13	0.2 (4)
C4—C5—C6—C7	178.5 (3)	C3—C11—C12—C13	−179.5 (3)
C5—C6—C7—C8	0.0 (5)	C11—C12—C13—C14	0.9 (4)
C6—C7—C8—C9	0.2 (5)	C12—C13—C14—C9	−0.6 (5)
C7—C8—C9—C14	178.9 (3)	C8—C9—C14—C13	179.7 (3)
C7—C8—C9—C10	−0.6 (5)	C10—C9—C14—C13	−0.8 (4)
C6—C5—C10—C11	178.8 (3)		

### Hydrogen-bond geometry (Å, °)

**Table:**

*D*—H···*A*	*D*—H	H···*A*	*D*···*A*	*D*—H···*A*
O1—H1A···O2^i^	0.82	1.97	2.771 (4)	165
C2—H2A···O2	0.97	2.31	2.714 (5)	104

Symmetry codes: (i) −*x*+1, −*y*+1, −*z*.
